# The challenge of comprehensively mapping children's health in a nation-wide health survey: Design of the German KiGGS-Study

**DOI:** 10.1186/1471-2458-8-196

**Published:** 2008-06-04

**Authors:** Bärbel-Maria Kurth, Panagiotis Kamtsiuris, Heike Hölling, Martin Schlaud, Rüdiger Dölle, Ute Ellert, Heidrun Kahl, Hiltraud Knopf, Michael Lange, Gert BM Mensink, Hannelore Neuhauser, Angelika Schaffrath Rosario, Christa Scheidt-Nave, Liane Schenk, Robert Schlack, Heribert Stolzenberg, Michael Thamm, Wulf Thierfelder, Ute Wolf

**Affiliations:** 1 Department of Epidemiology and Health Reporting, Robert Koch Institute, Seestraße 10, 13353 Berlin, Germany; 2 Charité Center 1 for Health and Human Sciences, Institute of Medical Sociology, Thielallee 47, 14195 Berlin, Germany

## Abstract

**Background:**

From May 2003 to May 2006, the Robert Koch Institute conducted the German Health Interview and Examination Survey for Children and Adolescents (KiGGS). Aim of this first nationwide interview and examination survey was to collect comprehensive data on the health status of children and adolescents aged 0 to 17 years.

**Methods/Design:**

Participants were enrolled in two steps: first, 167 study locations (sample points) were chosen; second, subjects were randomly selected from the official registers of local residents. The survey involved questionnaires filled in by parents and parallel questionnaires for children aged 11 years and older, physical examinations and tests, and a computer assisted personal interview performed by study physicians. A wide range of blood and urine testing was carried out at central laboratories. A total of 17 641 children and adolescents were surveyed – 8985 boys and 8656 girls. The proportion of sample neutral drop-outs was 5.3%. The response rate was 66.6%.

**Discussion:**

The response rate showed little variation between age groups and sexes, but marked variation between resident aliens and Germans, between inhabitants of cities with a population of 100 000 or more and sample points with fewer inhabitants, as well as between the old West German states and the former East German states. By analysing the short non-responder questionnaires it was proven that the collected data give comprehensive and nationally representative evidence on the health status of children and adolescents aged 0 to 17 years.

## Background

Several representative Health Interview and Examination Surveys have been carried out on the adult population in Germany [1].

However, no such study existed for the 0- to 17-year-old at the turn of the millennium. Available data on the health of children and adolescents included vital statistics, administrative medical data, records from routine medical check-ups of children starting school, and various regional epidemiological studies. This patchwork of data and results was of limited use for assessing the general health of the young generation in Germany. Due to differences in methods, instruments and definitions, sources yielded inconsistent or even contradictory results.

Therefore the German Federal Ministry of Health commissioned the Robert Koch Institute (RKI) to design and conduct a nationwide study on the health of the young generation. In 1998, the RKI began to develop strategies and instruments based on its extensive experience from health surveys of adults, on thorough literature reviews, and on close co-operation with other experts. After evaluation of the study protocol by reviewers and approval by ethics committees and data protection officers, a feasibility study was carried out in 2001–2002. A total of 1630 children and adolescents were enrolled and all logistics and instruments tested. This pre-test was sponsored by the German Federal Ministry for Research and Education. In the light of this pre-test, the study plan and all procedures were optimised for the main survey with some 18 000 subjects.

Study participants were enrolled from May 2003 to May 2006, all logistics and examinations were carried out by RKI staff. The study was monitored by a Scientific Advisory Board consisting of experts in the fields of epidemiology, paediatrics, health surveys, and ethics.

Design and methods of this first Health and Examination survey for children and adolescents in Germany are described in this paper.

## Methods/Design

### Sampling frame

The KiGGS survey is based on a nationally representative sample of children and adolescents 0–17 years of age with main residence in Germany. The sampling procedure was based on a two-stage protocol developed in co-operation with the Centre for Survey Research and Methodology (ZUMA), Mannheim, Germany. The study was approved by the Charité/Universitätsmedizin Berlin ethics committee and the Federal Office for the Protection of Data.

First, a systematic sample of 167 primary sample units (PSUs) was drawn from an inventory of German communities stratified according to the BIK classification system [2], which measures the grade of urbanization, and the geographic distribution. The number of PSUs per strata was determined using the Cox procedure for community sampling [3] with sampling probability proportional to population size. In order to ensure sufficient sample size for analyses stratifying according to residence in former East or West Germany, a disproportionate number of PSUs was included to represent former West (n = 112) and East (n = 50) Germany, and the city of Berlin (n = 5). At the second stage, an equal number of addresses (n = 24) per birth cohort were randomly selected (simple random sample) from local population registries within selected PSUs 8 weeks prior to the start of examinations. A final simple random sample was drawn at the Robert Koch Institute, including a total of 8, 9 or 10 children and adolescents per birth cohort, depending on community size. Thus, the target population per PSU consisted of 144, 162 or 180 persons eligible to be contacted and invited to participate in the study. Oversampling of children and adolescents from families with a migration nationality was used, as we expected a higher proportion of undeliverable contacts and non-respondents in this subgroup compared to children and youths from non-migrant families. The total KiGGS sample included 28,299 children and adolescents.

### Recruitment of the study population and field logistics

Parents of eligible children and adolescents were contacted by letter and invited to participate in the survey. Follwing a random route plan, 167 PSUs were covered by four study teams within three years (19 May 2003 to 6 May 2006). A number of strategies were applied to maximize participation. The start of the survey within communities was advertised by local media and community networks. Personal contact was sought to 14- to 17-year-olds and to families who had not responded to the invitation letter. In addition, small incentives were offered to increase participation. In order to increase participation of children with a migration background, invitation letters, information material and questionnaires were translated into six languages and migrant-specific public relation actions were targeted at the specific migrant population of the individual PSU.

### Modular Structure

**Figure 1:**
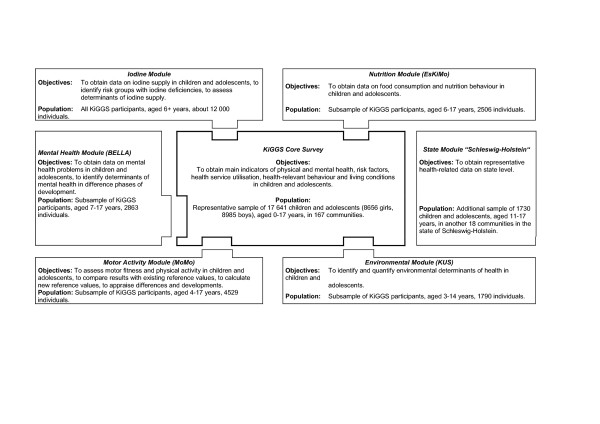
**Modular structure of the KiGGS survey**. Modular structure of the KiGGS survey.

This study consisted of the KiGGS core survey and five modules, as shown in Figure 1[4]. Four modules were in-depth studies carried out with subsamples of KiGGS participants and focussing on mental health, environmental exposures, motor fitness, and nutrition, respectively. The fifth module was an increase in sample size in the federal state of Schleswig-Holstein, in order to achieve a representative statewide sample. Here, we only report on the core survey.

### Health topics and instruments

**Figure 2:**
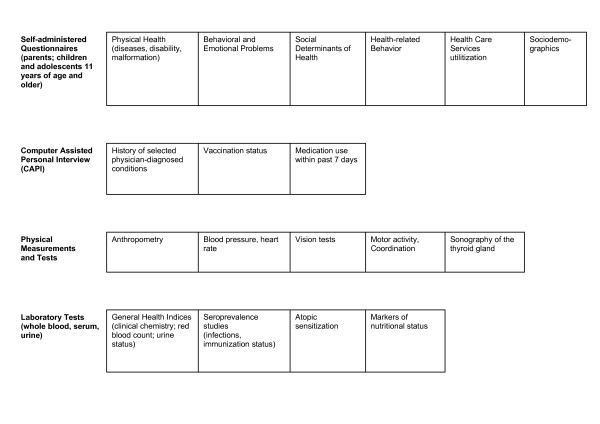
**Topics covered and instruments used in the KiGGS survey**. Topics covered and instruments used in the KiGGS survey.

The survey involved questionnaires filled in by parents and parallel questionnaires for children and adolescents from the age of 11 years onwards, physical examinations and tests, and a computer assisted personal interview (CAPI) performed by a physician. The topics covered by this interview are summarized in the Additional file 1. Testing of blood and urine samples was carried out at central laboratories. Figure 2 gives an overview of health-related information and objective measurements collected in the KiGGS study.

### Self-administered questionnaires

Self-administered parent questionnaires were designed age specifically considering developmental, health and health care issues relevant to children and young people of particular age groups (0–2, 3–6, 7–10, 11–13, 14–17 years of age). Apart from questions on sociodemographic variables, general somatic and mental health, sense of well-being, family structure, social environment and living conditions, self-administered questionnaires contained several specific items and instruments.

The screener for children with special health care needs (CSHCN) was part of the KiGGS questionnaire for parents. The CSHCN screener is a validated, 5-item survey-based measure for identifying CSHCN based on parent-reported consequences experienced by children with ongoing health conditions [5,6]. The screener identifies not only CSHCN who currently use health services but also CSHCN who may need services they are not receiving.

The Strengths and Difficulties Questionnaire (SDQ) was included as a brief screening questionnaire to assess emotional symptoms, conduct problems, hyperactivity/inattention, peer problems and, as a dimension of strength, prosocial behaviour. In the KiGGS study, the 25-item self-rated version was used for 11- to 17-year-olds as well as the 25-item version for completion by the parents of children aged 3–17 years [7,8].

The SCOFF 5-question screening questionnaire was used to detect symptoms of eating disorders by addressing core features of anorexia nervosa and bulimia nervosa [9,10]. The instrument is not a diagnostic tool, but defines risk groups.

Protective factors for mental health were examined in terms of personal, family and social resources. Among 14- to 17-year-olds, personal resources were assessed using the WIRKALL scale on self efficacy by Schwarzer & Jerusalem [11]. Additionally an in-house scale [12] of 5 items was used for children 11–17 years of age. This instrument comprised one item concerning optimism of the Berner Fragebogen zum Wohlbefinden Jugendlicher (BFW) [13], one item of the Children's Sense of Coherence Scale (CSOC) [14], and three items of the WIRKALL scale [11]. Family climate was assessed by a modified version of the Family Climate Scale according to Schneewind [15], social resources were assessed by the Social Support Scale (SSS) of Donald and Ware [16].

To measure health related quality of life (HRQOL) we used the internationally employed KINDL-R generic HRQOL questionnaire consisting of 24 items, which form a total score and six subscales (physical well-being, emotional well-being, self-esteem, family, friends and every day functioning, i.e. at school or nursery school/kindergarten). For all 3–17 year olds parents reported on their HRQOL and 11–17-year olds filled in the questionnaire themselves additionally [17].

Information on food intake was collected with a self-administered semi-quantitative food frequency questionnaire including 54 items [18]. Parents of children aged 1–10 years and participants aged 11–17 years received this questionnaire by mail several weeks before the health examination. About 95% of the questionnaires were returned.

### Operationalization of sociodemographic and migrant status

The parent questionnaire included standardized questions on both parents' educational and professional status as well as total income available to the family household, as recommended by the German Society of Epidemiology (Jöckel et al. 1998). Separate social status scores were computed from each of the three components. The sum of the scores was calculated ranging from a possible minimum of 3 to a maximum of 21. Scores were computed for each parent separately, and the higher score was used to define the child's social status. If parents were separated, the score of the main caregiver was used. Categories of social status were defined as lower (3–8), intermediate (9–14), and upper (15–21), as previously described (Winkler, Stolzenberg 1999).

The information collected on migration background included nationality, country of birth and year of immigration of both parents, categorisation to migrant groups by legal and historical criteria (e.g. labour migrants, asylum seekers etc), languages spoken at home and proficiency of the German language of both children and parents.

This allows a multidimensional view on the migration background including cultural and ethnic origin, migration background of both vs. only one parent, first generation migrants vs. descendants of migrants and legal status.

### Computer-assisted Personal Interview (CAPI)

Using a CAPI, specifically trained study physicians obtained detailed information on the participating children's and adolescents' medical history from accompanying parents or caregivers. The interview covered a selected number of physician-diagnosed chronic conditions (hay fever, atopic dermatitis, asthma, obstructive bronchitis, pneumonia, otitis media, heart disease, anaemia, seizure, thyroid problems, diabetes, scoliosis, migraine). Parents were asked whether the respective condition had been diagnosed and whether it had been "present in the past 12 months". The CAPI also comprised a detailed interview on any medication use (prescription as well as over-the-counter drugs) within the 7 days preceding the interview. In the invitation, parents had been asked to bring original containers or package inserts to the examination site for verification. It was asked whether the medicine had been prescribed by a doctor, for what reasons and how often the product was taken, whether the child's condition had improved or whether there had been any adverse drug reactions or side-effects. Specific ATC (Anatomical Therapeutic Chemical) codes were assigned to all reported medications [see additional file 1].

### Measurements

Standardized anthropometric measures were obtained, including body height and weight, humerus length, triceps and subscapular skinfold thickness, and head, waist and hip circumferences [19,20]. Body height, lengths and circumferences were measured to the nearest 0.1 cm using calibrated infantometers or stadiometers (Holtain Ltd., UK), and flexible, non-stretchable measuring tapes (Siber Hegner, Ltd., Switzerland). Triceps and subscapular skinfold thickness were assessed to the nearest 0.2 mm with a Harpenden caliper (Holtain Ltd., UK). Electronic scales (SECA, Ltd., Germany) were used to measure body weight to the nearest 0.1 kg. From these measures we calculated the waist-to-hip ratio, the body mass index (BMI), body fatness according to Slaughter equations [21], and the frame index of growth and nutritional status [22].

Among 10- to 17-year-old participants, pubertal stage was assessed based on menarchal status (premenarchal or postmenarchal) or voice change and self-estimated Tanner stage of pubic hair growth patterns according to standardized drawings [23]. Standardized measures of heart rate and blood pressure were obtained from participants 3–17 years of age using an automated device (Datascope Accutorr Plus). Two independent readings of systolic, diastolic and mean arterial pressure and one reading of heart rate were taken in an upright position after 5 minutes of rest. Thyroid sonography was performed among children and adolescents 6 years of age and older (ROI USD 200 Sirius, 6/8 MHz, ROI Medical Systems, Germany).

### Laboratory tests

Casual urine specimens were obtained and casual venous blood samples were taken with evacuated EDTA and gel tubes. The time of blood collection and hours since last food intake were documented. Blood specimens were processed within 45 minutes according to a highly standardized protocol [24,25]. Whole blood was kept in the original EDTA collection tubes at 4°C except for a 100 μl aliquot; this was hemolyzed under light protection and stored at -50°C for analysis of folic acid. Serum was stored at -50°C in 500 μl aliquots as available. Urine samples were divided into two aliquots (1 ml, 10 ml) and kept frozen at -50°C. Specimens were sent by car for central analysis or storage in Berlin within an average of 18 hours. Serum specimens were shipped on frozen cool packs, EDTA blood at 4°C. Automated analyses of routine biochemical measures, such as red blood count and clinical chemistry were performed in a university hospital laboratory (Deutsches Herzzentrum Berlin). Parameters requiring time consuming preparation and standardization of laboratory methods were processed in an epidemiological research laboratory (Robert Koch Institute, Berlin). Analyses were subject to rigorous internal and external quality control according to guidelines of the German Medical Association [26]. Quality control measures were monitored and evaluated under the auspices of the Robert Koch Institute. Laboratory methods have previously been described in detail [27]. An overview of biochemical measurements, laboratory methods and the specific tests and equipment used is provided in the Additional files 2 and 3.

### Non-response questionnaire

Parents who declined participation were asked to fill out a brief 15-item questionnaire, in order to provide basic sociodemographic and health-related information about the family and child for non-response analysis.

### Data management and preparation for analysis

During the 3-year data collection phase, approx. 1500 items were recorded for every study subject. Data management was not limited to survey data collection and administration, but also comprised the provision of tools for a highly standardized and partly automated control of process data. These measures provided a validated and cleaned final data set and detailed documentation within less than 6 months after the completion of data collection.

In order to assure that estimates derived from the KiGGS study are representative at the national level, survey weights were developed to be applied throughout statistical analyses. This included weighting for sampling design as well as weighting to adjust for deviations between the design-weighted net sample and German population statistics (as of 31 December 2004) based on cross-classifications by age, sex, residence in Western or Eastern Germany, and nationality (German vs. non-German). Weighting mainly resulted in correction for differences in age structure and disproportionately higher sample size in Eastern vs. Western Germany.

### Quality assurance

To achieve a high degree of standardisation in the survey, the examination teams were initially trained and then underwent continuous supervision. The quality management covered internal and independent external quality control measures monitoring each step of data collection and data processing as well as the training sessions. Quality standards and requirements for internal quality control were documented in the operation manual following recognized epidemiologic guidelines. External quality assurance was carried out by the Institute of Epidemiology at the GSF National Research Centre for Environment and Health as an audit of internal quality assurance as well as systematic observation and spot checks to ensure quality requirements were fulfilled.

### Response

Table 1 summarizes response proportions by main sociodemographic variables and reasons for non-response. The overall percentage of non-deliverable survey contacts was 5.3%, leaving a target population of N = 26 787. At an overall response rate of 66.6%, the final study population included 17 641 children. Response proportions did not materially differ by gender or age groups, but were significantly lower among children from families with a migration background compared to native German families. Response was also significantly lower in larger cities (≥ 100 000 residents) than in smaller communities.

**Table 1:** Response rates and reasons for non-response

	**N**	**%**
**Total sample size**	28 299	
**Undeliverable survey contacts**	1512	5.3
Address unknown		49.0
Moved to unknown place		20.7
Language problems		18.7
Main residence outside PSU		11.5
Deceased		0.1
**Target population**	26 787	

**NONRESPONSE**	**N**	**%**

**Non-responders**	9146	
No-shows, cancellations		6.0
Withdrawn (child scared to participate)		5.7
Not interested (healthy child)		4.2
Refused		3.5
No time		3.2
Unable to reach		2.6
No suitable appointment		2.0
Child needs continuous medical care		1.3
Too much effort; intolerable for child		1.1
Not a place of residence		0.8
Health problems		0.5
Other reasons		2.5

**STUDY PARTICIPANTS, RESPONSE**	**N**	**%**

**Total study population**	**17 641**	**66.6**
**Gender**		
Girls	8656	67
Boys	8985	66
**Age groups (years)**		
0–2	2805	65
3–6	3875	66
7–10	4148	70
11–13	3076	69
14–17	3737	63
**Citizenship**		
Non-German	1479	51
German	16 162	68
**Region of residence**		
Western Germany	11 741	65
Eastern Germany	5362	70
Berlin	538	61

### Non-response analysis

The most common reason for non-response was unexplained absence or late cancellation shortly before the appointment (Table 1). No-shows and missed appointments are problematic for the organisation, but could be compensated by a short-term arrangement of appointments with participants who had been placed on a waiting list.

Since key socio-demographic and health-related characteristics for children and parents could be registered for two-thirds of the non-respondents, basic information is available for 89% of the target population and could be compared between responders and non-responders. The educational data suggests a slight middle-class bias, but there are hardly any differences for the health-related variables between the two groups. For example, the general health of the participants (93.7% very good and good) and non-respondents (92.5% positive) was assessed equally by the parents.

## Discussion

The German Health Interview and Examination Survey for Children and Adolescents (KiGGS) collected comprehensive data on the health status of children and adolescents living in Germany aged 0 to 17 years. This survey is unique in Europe by its sample size, its range of age and its response rate, as it can be seen by the EU Health Surveys Information Database.

The main aims of this survey were

• To close information gaps in the field of children's and adolescents' health,

• To obtain data for epidemiological research,

• To obtain baseline data for longitudinal epidemiological studies in children and adolescents,

• To evaluate the effectiveness of the health care system for the young generation

• To help prioritising public health measures,

• To give recommendations to health policy makers for improving prevention and intervention strategies,

• To improve the long-term health of children and adolescents.

Most of these aims are reached. The data will be analysed in a timely and systematic manner by the Robert Koch Institute. Initially, mainly descriptive analyses will provide information on the distribution of main health characteristics according to sociodemographic key variables including age, sex, region of residence (former East/West Germany), social status, and migration background. In further analyses conclusions will be drawn for setting priorities in improving long-term health of children and adolescents and to give recommendations to health policy makers for improving prevention and intervention strategies in Germany.

Data from the KiGGS survey will be made available to the scientific community as a public use file by the end of 2008. In the meantime, there is the opportunity to use KiGGS data by research co-operation with the RKI in order to jointly answer defined scientific questions. All necessary information has been compiled for co-operation partners.

## Competing interests

The authors declare that they have no competing interests.

## Authors' contributions

B–MK, PK, HH, MS conceptualized and supervised the study and provided assistance in analyzing the data, interpreting the results and writing the final manuscript. UE, HKahl, HKnopf, GBMM, LS, WT, MT, ML, UW, HN, CS–N provided specific knowledge, assisted in the conceptualization of the study and made substantial contributions to the acquisition and quality assurance of data as well as writing of the final manuscript. ASR, HS, RD provided specific knowledge, contributed to interpreting the results and writing the final manuscript and were responsible for data management and analysis. All authors read and approved the final manuscript.

## Pre-publication history

The pre-publication history for this paper can be accessed here:



## Supplementary Material

Additional file 1 **The Computer-assisted Personal Interview (CAPI)**. The Computer-assisted Personal Interview (CAPI). Click here for file

Additional file 2 **Laboratory measurements concerning the topics „Nutrient Deficiencies”, „Disease Indicators” and "Risk Factors associated with Non-Communicable Diseases"**. Laboratory measurements concerning the topics „Nutrient Deficiencies”, „Disease Indicators” and "Risk Factors associated with Non-Communicable Diseases". Click here for file

Additional file 3 **Laboratory parameters concerning „Seroprevalence and Immunization Status“**. Laboratory parameters concerning „Seroprevalence and Immunization Status“. Click here for file
