# Supplementary material for: The challenge of comprehensively mapping children's health in a nation-wide health survey: Design of the German KiGGS-Study
Source: BMC Public Health. 2008 Jun 4;8:196. doi: 10.1186/1471-2458-8-196 (PMC2442072; doi:10.1186/1471-2458-8-196)
Supplement: Additional file 1 — The Computer-assisted Personal Interview (CAPI). The Computer-assisted Personal Interview (CAPI). [file 1471-2458-8-196-S1.doc]

| **Topic** | **Instrument** | **Operationalisation** |
| --- | --- | --- |
| hay fever | CAPI | doctor-diagnosed |
| atopic dermatitis | CAPI | doctor-diagnosed |
| asthma | CAPI | doctor-diagnosed |
| obstructive bronchitis | CAPI | doctor-diagnosed |
| pneumonia | CAPI | doctor-diagnosed |
| otitis media | CAPI | doctor-diagnosed |
| heart disease | CAPI | doctor-diagnosed |
| anaemia | CAPI | doctor-diagnosed |
| seizure | CAPI | doctor-diagnosed |
| thyroid problems | CAPI | doctor-diagnosed |
| diabetes | CAPI | doctor-diagnosed |
| scoliosis | CAPI | doctor-diagnosed |
| migraine | CAPI | doctor-diagnosed |
| any allergy testing and result | CAPI | doctor-diagnosed |
| surgical operations | CAPI | doctor-diagnosed |
| immunization | vaccination certificate | Completeness and timeliness, according to recommendations in Germany |
| any reasons for not having the child immunised | CAPI |  |
| vaccination side effects | CAPI |  |
| medication, dietary supplements | CAPI | Taken within the past 7 days |
