# Supplementary material for: The challenge of comprehensively mapping children's health in a nation-wide health survey: Design of the German KiGGS-Study
Source: BMC Public Health. 2008 Jun 4;8:196. doi: 10.1186/1471-2458-8-196 (PMC2442072; doi:10.1186/1471-2458-8-196)
Supplement: Additional file 2 — Laboratory measurements concerning the topics „Nutrient Deficiencies”, „Disease Indicators” and "Risk Factors associated with Non-Communicable Diseases". Laboratory measurements concerning the topics „Nutrient Deficiencies”, „Disease Indicators” and "Risk Factors associated with Non-Communicable Diseases". [file 1471-2458-8-196-S2.doc]

| **Analyte (Unit)** | **Method** | **Equipment** |
| --- | --- | --- |
| Folic acid in serum [ng/ml] | ECLIA*^1^, Folate I | Elecsys E 2010 |
| Folic acid in blood [ng/ml] | ECLIA, Folate I | Elecsys E 2010 |
| Folic acid in serum [ng/ml] | ECLIA, Folate II | Elecsys E 2010 |
| Folic acid in blood [ng/ml] | ECLIA, Folate II | Elecsys E 2010 |
| Vitamin B12 [ng/l] | ECLIA | Elecsys E 2010 |
| Iron [µmol/l] | Ferrocene method without deproteination (Roche, Mannheim) | Hitachi 917 |
| s-TfR [mg/l] | Enhanced latex agglutination assay (Behring, Marburg) | BNA- nephelometer (Behring) |
| Ferritin [µg/l] | ECLIA | Elecsys E 2010 |
| Erythrocyte count (RBC) [10^6^/ml] | Impedance | Cell-Dyn 3500 (Abbott, Wiesbaden), |
| Leukocyte count (WBC) [k/µl] | Impedance | Cell-Dyn 3500 |
| Hemoglobin in blood (Hb) [g/dl] | Photometry | Cell-Dyn 3500 |
| Hematocrit (PCV) [%] | Calculated parameter | Cell-Dyn 3500 |
| Mean corpuscular haemoglobin (MCH) [pg] | Calculated parameter | Cell-Dyn 3500 |
| Mean corpuscular hemoglobin concentration (MCHC) [g/dl] | Calculated parameter | Cell-Dyn 3500 |
| Mean corpuscular volume (MCV) [fl] | Calculated parameter | Cell-Dyn 3500 |
| Cholesterol- total [mg/dl] | Enzyme assay (cholesterol-oxidase-PAP method) (Roche, Mannheim) | Hitachi 917 |
| HDL- Cholesterol [mg/dl] | Homogeneous enzymatic colorimetric assay (Roche, Mannheim) | Hitachi 917 |
| LDL- Cholesterol [mg/dl] | Homogeneous enzymatic colorimetric assay (Roche, Mannheim) | Hitachi 917 |
| Triglycerides [mg/dl] | Total glycerol with GPO-PAP- reaction (Roche, Mannheim) | Hitachi 917 |
| Homocysteine [µmol/l] | Fluorescent particle immunoassay (Abbott) | Axsym (Abbott) |
| Uric acid [mg/dl] | Uricase-PAP- reaction (Roche, Mannheim) | Hitachi 917 |
| C-reactive protein (CRP-S) [µg/l] | Immunological precipitation assay (until June 2004 SCIL, Martinsried) | Hitachi 917 |
| C-reactive protein (CRP-S) [µg/l] | Immunological precipitation assay (since July 2004 Roche) | Hitachi 917 |
| Glucose [mg/dl] | Hexokinase method(Roche, Mannheim) | Hitachi 917 |
| Glucose in urin | Combur9 |  |
| HbA1c [%] | High performance liquid chromatography (HPLC) | Diastat (Bio-Rad, München) |
| TSH [µU/ml] | ECLIA | Elecsys E2010 |
| Free T3 (fT3) [pg/ml] | ECLIA | Elecsys E2010 |
| Free T4 (fT4) [pg/ml] | ECLIA | Elecsys E2010 |
| Iodine in urin [µg/l] | Photometry (kinetics) after Sandell-Kolthoff (1937), ammonium peroxodisulfate- disintegration (Pino et al. 1996) | Cobas Mira Plus (Roche, Grenzach-Whylen) |
| 25(OH)Vitamin D [nmol/l] | Enzyme immunoassay | MTP-Reader ATTC 340 |
| 25(OH)Vitamin D [nmol/l] | Luminescence immunoassay (LIA) (DiaSorin, Diezenbach) | Liaison (DiaSorin, Diezenbach) |
| Parathormone [pmol/l] | Luminescence immunoassay (LIA) (DiaSorin, Diezenbach) | Liaison (DiaSorin, Diezenbach) |
| Calcium [mmol/l] | ortho-cresol phthalein- complexon method (Roche, Mannheim) | Hitachi 917 |
| Anorganic phosphate [mmol/l] | Ammonium-phosphomolybdate method (Roche, Mannheim) | Hitachi 917 |
| Alkaline phosphatase [U/l] | Optimized IFCC*^2^ standard method (Roche, Mannheim) | Hitachi 917 |
| Gamma glutamyl transferase (GGT) [U/l] | Optimized IFCC method (Roche, Mannheim) | Hitachi 917 |
| Potassium [mmol/l] | Potentiometry with ion selective electrode (Roche, Mannheim) | Hitachi 917 |
| Magnesium | Xylidyl blue assay (Roche, Mannheim) | Hitachi 917 |
| Total protein [g/dl] | Biuret assay (Roche, Mannheim) | Hitachi 917 |
| Urine creatinine (spot urine) | Jaffé method without deproteination (Roche, Mannheim | Hitachi 917 |
| Specific IgE | Fluorescence immunoassay (CAP, Phadia) | UNICAP 1000 |

*^1^ ECLIA: Electrochemiluminescence- Immunoassay

*^2^ IFCC: International Federation of Clinical Chemistry and Laboratory Medicine
