# Supplementary material for: The challenge of comprehensively mapping children's health in a nation-wide health survey: Design of the German KiGGS-Study
Source: BMC Public Health. 2008 Jun 4;8:196. doi: 10.1186/1471-2458-8-196 (PMC2442072; doi:10.1186/1471-2458-8-196)
Supplement: Additional file 3 — Laboratory parameters concerning „Seroprevalence and Immunization Status“. Laboratory parameters concerning „Seroprevalence and Immunization Status“. [file 1471-2458-8-196-S3.doc]

| **Analyte (unit)** | **Method (Manufacturer)** | **Device** | **Cut off** |  |
| --- | --- | --- | --- | --- |
| HSV1/HSV2- IgG (qualitative) | Enzyme immunoassay (Focus Diagnostics, Cypress, USA) | ATT340 Reader | <0,7  <1,3 | Neg.  BL* |
| Helicobacter pylori IgG (qualitative) | Enzyme immunoassay (Genzyme Virotech) | ATT340 Reader | >11 | Pos. |
| Anti-HAV [IU/ml] | ECLIA (Roche) | Elecsys E2010 | ≥20 | Pos. |
| Anti-HBs [mIU/ml] | ECLIA (Roche) | Elecsys E2010 | >10 | Pos. |
| Anti-HBc (qualitative) | ECLIA (Roche) | Elecsys E2010 | <1 | Pos. |
| HBsAg (qualitative) | ECLIA (Roche) | Elecsys E2010 | >1 | Pos. |
| Measles antibodies [IU/ml] | Enzyme immunoassay (Behring) | Tecan Spectral Fluor plus | <150  ≤300 | Neg.  BL |
| Mumps antibodies (titer) | Enzyme immunoassay (Behring) | Tecan Spectral Fluor plus | <1:320  ≤1:500 | Neg.  BL |
| Rubella antibodies [IU/ml] | Enzyme immunoassay (Behring) | Tecan Spectral Fluor plus | <4  ≤7 | Neg.  BL |
| Polio antibodies (serum dilution) | Neutralization assay (in house) | Microscopy | <1:4 | Neg. |
| EHEC antibodies (titer) | Mikro-Widal (in house) | - | <1:160 | Neg. |
| Salmonella antibodies (titer) | Enzymimmunoassay (in house) | Labsystems Multiscan Ascent | <1:200  <1:400 | Neg.  BL |

* BL: borderline
